# Dissecting the Physiology and Pathophysiology of Glucagon-Like Peptide-1

**DOI:** 10.3389/fendo.2018.00584

**Published:** 2018-10-11

**Authors:** Silvano Paternoster, Marco Falasca

**Affiliations:** Metabolic Signalling Group, School of Pharmacy and Biomedical Sciences, Curtin Health Innovation Research Institute, Curtin University, Perth, WA, Australia

**Keywords:** glucagon-like peptide-1, metabolic disease, type 2 diabetes, enteroendocrine cell system, GPCR, L-cells, microbiome, α-cells

## Abstract

An aging world population exposed to a sedentary life style is currently plagued by chronic metabolic diseases, such as type-2 diabetes, that are spreading worldwide at an unprecedented rate. One of the most promising pharmacological approaches for the management of type 2 diabetes takes advantage of the peptide hormone glucagon-like peptide-1 (GLP-1) under the form of protease resistant mimetics, and DPP-IV inhibitors. Despite the improved quality of life, long-term treatments with these new classes of drugs are riddled with serious and life-threatening side-effects, with no overall cure of the disease. New evidence is shedding more light over the complex physiology of GLP-1 in health and metabolic diseases. Herein, we discuss the most recent advancements in the biology of gut receptors known to induce the secretion of GLP-1, to bridge the multiple gaps into our understanding of its physiology and pathology.

## Introduction

The gastrointestinal (GI) tract is a complex organ that monitors the body's energetical state and provides it with water and macro and micronutrients extracted from the ingested food. Along its length, the enteroendocrine cells (EECs) constitute a complex endocrine organ that communicates with the central nervous system (CNS) and the enteric nervous system (ENS) to orchestrate the homeostatic balance of the body in response to the GI luminal content.

This enteroendocrine system has traditionally been divided into 12 different cell types, based entirely on their hormonal content and cellular morphology. This endocrine organ is not organized in a glandular structure; on the contrary, it is dispersed heterogeneously, mainly as single cells, along the epithelium of the GI tract, from the stomach to the rectum with a defined cephalocaudal, crypt-to-villus in the small intestine and crypt-to-surface distribution in the colon (1, 2).

Despite representing just 1% of the adult gut epithelium, in the last decade it has become clear that the EECs constitute the largest endocrine organ in mammalia (3). Recent analysis of the expression of specific hormones at the cellular level, demonstrated that the EECs subdivision introduced above is outdated. Each enteroendocrine cell co-secretes multiple hormones with spatio-temporal, crypt-to-villus, and rostro-caudal variability, leading to the formation of overlapped gradients of individual hormones along the GI tract; the concept of well-defined subclasses of cells committed to express a specific subset of hormones independent of their location is currently untenable, thus detailed description of the topographical location of the cells needs to be implemented for future clarity (4).

Collectively, the EECs are responsible for the production of more than 30 different hormones that help to orchestrate the fate of the intermediary metabolism; acting upon different organs such as the pancreatic islets, the hypothalamus or the stomach, for the release of insulin, to regulate food intake or gastric emptying respectively (5–8).

Surprisingly, this heterogeneous and highly plastic population of cells is known to differentiate from a single staminal progenitor that gives also rise to enterocytes, goblet and paneth cells (1, 9).

It has been known for more than a century that the gut is capable to stimulate the endocrine portion of the pancreas and even improve the hyperglycaemic state of diabetic patients (10, 11). In 1932, the Belgian investigator LaBarre referred to these “factors” extracted from the intestinal mucosa as “incrétine,” deriving it from: INtestinal seCRETion of insulin (12). In the 60s, different authors demonstrated that oral glucose was capable to induce a 2-fold increase in insulin compared to an in-vein isoglycaemic administration (13).

In the last three decades, the incretin-effect has been attributed primarily to two peptide hormones, the gastric-insulinotropic peptide (GIP) and glucagon-like peptide-1 (GLP-1), excreted primarily by duodenal (K) and ileo-colonic (L) enteroendocrine cells respectively (14). Indeed, type 2 diabetes (T2D) is a metabolic disease reported to involve an impaired intestinal release of GLP-1 and its co-secreted peptides oxyntomodulin and glicentin (15–17), together with an insulinotropic resistance to GIP in the pancreas (18) which lead to a deficient incretin system, purportedly causing the disease (19, 20). Despite being still largely unknown how hyper caloric diets are disrupting the incretin signaling, some authors have shown that even circadian rhythms disruption, and the saturated fat palmitate, are significant stressors capable to hamper GLP-1 secretion (21, 22).

Obesity and Type 2 diabetes are chronic diseases for which the most effective treatment is bariatric surgery. These invasive gut surgical procedures, aimed to reduce absorptive surface area of the proximal GI tract, such as Roux-en-Y gastric by-pass (RYGB) or Sleeve Gastrectomy (SLG), are associated with an improved glycaemic control, weight loss, and often with complete remission from T2DM (23).

Despite this, the complete remittance of a great fraction of RYGB patients represents a fascinating new case-series that points at the importance of the EECs and its modulation of the whole-body metabolism (24). As such, the study of this complex endocrine organ, might help us to create new pharmacological tools to amend the specific molecular axis that drive T2DM and the associated co-morbidities known to affect the cardiovascular (25, 26) and renal system (27, 28).

A panoply of contradictory studies have attempted to establish what is the possible role of GLP-1 or other gut peptides in the rapid, and long-lasting remittance from T2D after bariatric surgery, but no consensus about the identity of the molecular players has yet been reached (29–39).

Since 2005, there are on the market only two classes of drugs that attempt to bolster glucagon-like peptide-1 signaling, GLP-1 receptor agonists and DPP-IV inhibitors, for a supra-physiological GLP-1 activity. Unexpected safety-issues and important side-effects (40) prove that the peripheral hijack of this peptide is not sufficient, and does not replicate the remittance seen in bariatric surgery.

This review summarizes the most recent studies that reframe our understanding of the physiology of GLP-1 in health and disease.

## Chemosensation in GLP-1-producing cells

Intestinal proglucagon expressing cells were historically named L-cells more than 4 decades ago because of their large 500 nm secretory granules seen under electron microscopy (41). Today, we know that these are nutrient-responsive enteroendocrine cells that secrete a variety of peptide hormones, primarily derived from the proglucagon gene (*GCG*) (42). Once translated, the 180 amino acid long GCG protein is processed by two proteases, Psck1 and Psck3, to give GLP-1, GLP-2 but also the less studied and understood glicentin and oxyntomodulin (43). Other peptide hormones, such as insulin-like peptide 5 (INSL5) (44, 45), PYY (46), GIP and neurotensin (17, 47) can be co-expressed with the *GCG* products depending on the topographical localization of the cell; surprisingly, it appears that GLP-1 and PYY can be excreted independently possibly due to the existence of compartmentalized secretory vesicles (48).

**Figure 1:**
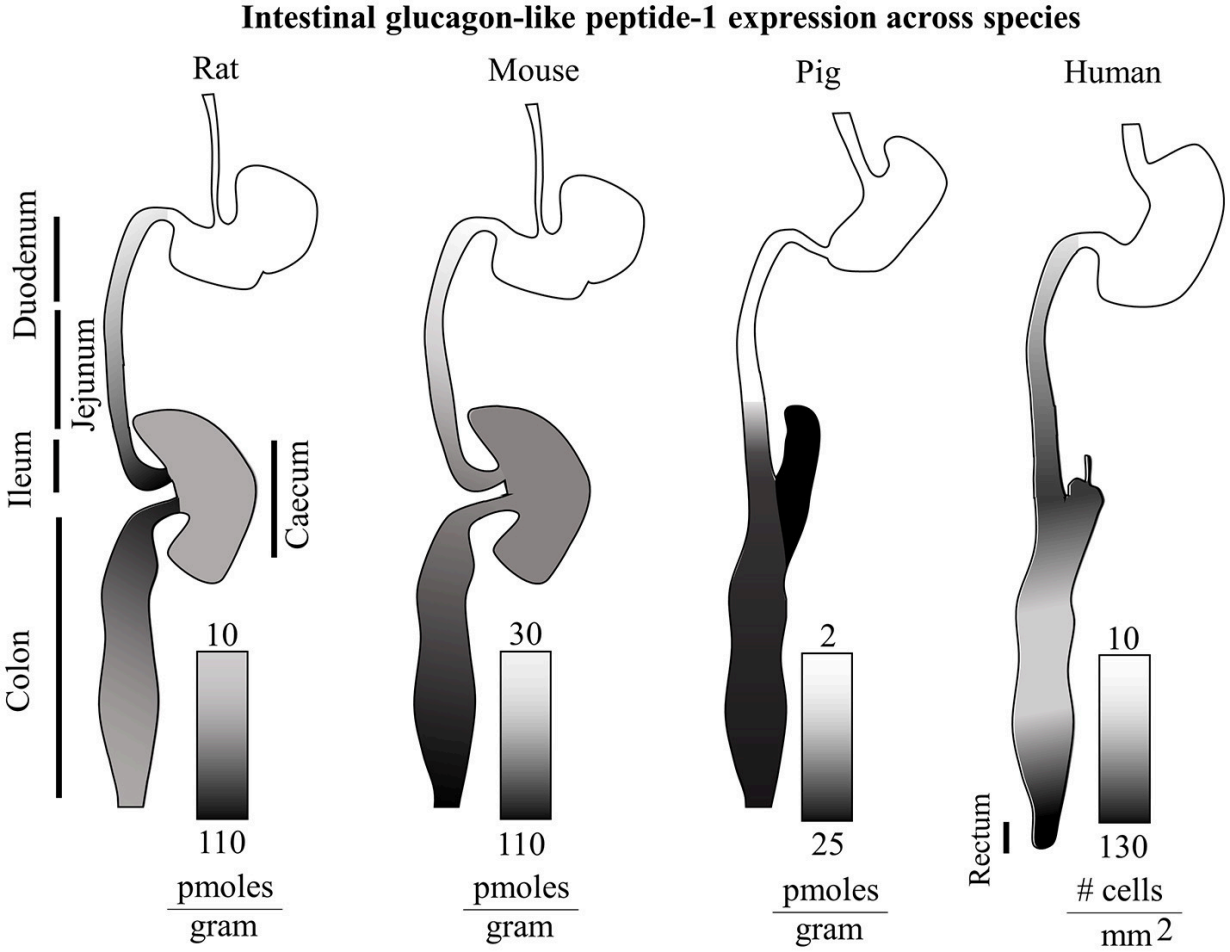
Intestinal glucagon-like peptide-1 expression across species. Total GLP-1 expression along the rat, mouse, pig and human intestinal tracts (relative lengths not to scale) is displayed with gradients as individually indicated in figure. The rat GI tract shows the highest levels of GLP-1 in the ileum and proximal colon. On the other hand the murine gut, displays the highest GLP-1 levels in the distal colon. The porcine intestine shows highest levels in the caecum and distal colon, and virtually none in the proximal small intestine. In humans, a steady increasing gradient along the small intestine is followed by a decrease in expression in the colon, and a second steeper gradient culminating in the rectum with the highest GLP-1 expression (49–51).

There appear to be considerable species-specificity in terms of anatomical localization of GLP-1 production as summarized in Figure 1. Independently of other hormones, in mice the distal colon and rectum show the higher levels of GLP-1 per gram of tissue. Conversely, in rats the distal ileum and in pigs the caecum are the anatomical regions with the highest amounts of GLP-1 (49). In humans, the density of GLP-1 and PYY positive cells increase steadily along the small intestine, decreasing in the colon, and then raising again reaching a maximum density in the rectum with the highest values of around 150 GLP-1-expressing cells per square millimeter. Curiously in type 2 diabetes, an equally distributed gradient of GCG and PC1/3 mRNA appears upregulated, but with normal GLP-1^+^ cell densities, indicating a possible translational resistance (51).

The L-cells derived cocktail of hormones is believed to play pivotal roles in digestion, for example slowing down the GI motility (PYY) and suppressing the appetite *in vivo* (GLP-1, oxyntomodulin, PYY), apparently in response to direct sensing of the gut luminal content via G-protein coupled receptors or through neuronal circuits (43, 52).

Current *in vitro* technologies are not capable to support for long-term *ex vivo* the growth of isolated GLP-1 producing-cells. The available knowledge about the biology of GLP-1 is primarily drawn upon studies operated with the murine-derived GLUTag or STC-1, and the human-derived NCI-H716 cell lines. It is important to understand that these *in vitro* models express a different hormonal cocktail and respond to different chemical stimuli than intestinal L-cells *in vivo* (53, 54). Primary cultures are another useful short-term system; nonetheless GLP-1-producing cells amount to only 1–2% of the whole cultured mucosal population, with considerable intra and inter-assay variability (53).

The more physiologically relevant studies make use of *in vivo* transgenic mice, *ex vivo* perfused intestines or, more recently, crypt organoids derived from human, mouse or porcine guts (55).

*In situ* immunostaining and FACS studies have demonstrated that the hormonal secretome of GLP-1-secreting-cells is anatomically dependent. In the upper gut where these cells are more sparse and rare, GLP-1 is co-expressed with GIP, a K-cell feature, but also with cholecystokinin (CCK) and Neurotensin (NT). Conversely in the colonic mucosa, GLP-1 co-localizes with PYY, CCK and the orexigenic Insulin-Like peptide 5 (INSL5) (4, 43, 45, 53, 56, 57). Interestingly, colonic L-cells possess twice as much total GLP-1 compared to L-cells from the upper GI tract (53). Furthermore, considering the differential response to glucose, it is clear that the physiology of this population of EECs is distinct, and evolved under a different evolutionary pressure dictated by the exposure to a different luminal content (53, 58).

L-cells are known to modulate the release of their hormonal cargo in response to the activation of a plethora of receptors capable to sense fats, carbohydrates, proteins and many other compounds. Enteroendocrine cells, like other endocrine, muscle and neuronal cells, are electrically excitable. Membrane depolarization, triggered by a ligand-bound receptor, results in a spike of intracellular calcium (Ca^2+^) which leads to the fusion of the endocrine granules with the lateral and the broader basal side, resulting in the discharge of a hormonal cargo in the capillaries of the mucosa.

Surprisingly, the EECs in the colon have been demonstrated to physically connect through a basal process named Neuropod, with afferent nerve cells residing in the lamina propria, defining a neuroepithelial circuit that expands the physiology of these cells (59). In fact, the idea of a direct neuronal regulation has been demonstrated decades ago in rats, where a bilateral vagotomy massively downregulates circulating PYY and GLP-1 levels after a glucose load (60). Furthermore, intracerebral acute, but not chronic administration of GLP-1 in mice, improves pancreatic glucose stimulated insulin secretion (61).

## GPCRs as molecular tastants

G-protein coupled receptors (GPCRs) are evolutionary ancient proteins spanning seven times across the plasma membrane of virtually any known cell type. In metazoans, these proteins evolved into thousands different molecular transducers capable to translate the presence of extracellular molecules into intracellular cascades of messages amplified by different G-proteins, which in turn enforce a myriad of different cellular processes via secondary messengers (62). The transmembrane domain of these chemosensors being exposed to a tighter evolutionary pressure lead to a relative evolutionary stability of the same 3-dimensional structure. On the contrary, the extracellular facing portion is what primarily defines the identity of a myriad of different receptors, capable to sense a panoply of molecular entities ranging in size from a single atom to hundreds aminoacids long proteins. The intracellular portion of these nano-sensors, has evolved in humans in a complex hub that triggers multiple molecular cascades that results in short-term and long-term modifications of the target cell and even the whole-body metabolism.

Different receptors, expressed by the same cell type or tissue, can trigger the same molecular cascade. With this notion, the study of these molecular transducers has been approached by some authors in recent years from a top-down point of view, whereby sub-type specific, allosteric positive or negative modulators (PAM, NAMs), as well as direct agonists, are utilized as tools for pathway dissection and analysis (63, 64). In the last decade, technological advancements in techniques such as circular dichroism (65), Cryo-electron microscopy (Cryo-EM) (66) and crystallography (67) have expanded our understanding of the physiology of multiple chemosensors expressed by L-cells, which led to the discovery of new molecular tools with possible future clinical applications in diseases such as type 2 diabetes (64, 68–70).

The expression of different GPCRs to restricted anatomical regions, such as the enteroendocrine cell system, is a finely tuned system that evolved in metazoan. Macronutrients, bile acids (BAs), and microbiota-derived compounds activate many of these GPCRs expressed by GLP-1 expressing cells (71). Nonetheless, not all intestinal stimuli signals through these chemosensors; for example glucose induces the release of GLP-1 from human duodenum and ileum via electrogenic transporters (SGLT1) and voltage-gated Calcium and Sodium channels responsible for the membrane depolarization and hormonal release (53, 72).

The main G protein-coupled receptors which activation appears to cause the release of GLP-1 are: GPRC6A (73), GPR40-41-42-43-93-119-120 (43), GPR142, GHS-R1A (74), Tas1R2-Tas2R3(T1R2-T1R3) (75), GPBAR1 (TGR5), and CasR (6, 76, 77) (Table 1). The functional differences seen between Jejunum-Ileal and colonic GLP-1 producing cells, could be explained by a different pool of GPCRs, or possibly by the presence of heteromers displaying a more complex pharmacology than with each individual receptor.

**Table 1:** Demonstrated primary effects of the major GLP-1-stimulating receptors.

**Receptor**	**Ligand**	**Effect**	**Experimental condition**	**References**
FFAR1/GPR40	Palmitate	Insulin ↑, glucagon ↑, somatostatin↑	*Ex-vivo* human islets	(78)
	Free fatty acids	GLP-1 ↑, GIP ↑	*In-vivo* mouse	(79)
	Long chain fatty acids	CCK ↑	*Ex-vivo* murine duodenal I cells	(80)
FFAR2/GPR43	Inulin	PYY ↑	*In-vivo* diabetic mouse	(81)
	Propionate	PYY ↑, GLP-1 ↑	*Ex-vivo* murine colonic Primary cultures, & *in-vivo* murine and rat	(82)
FFAR3/GPR41	Propionate	PYY ↑, GLP-1 ↑	*Ex-vivo* murine colonic primary cultures	(83)
FFAR4/GPR120	α-Linolenic acid	GLP-1 ↑	*In-vivo* mouse	(84)
	Lard oil, corn oil	GIP ↑, CCK ↑	*In-vivo* mouse	(85, 86)
GPR119	Oleoyl-LPI, OEA	GLP-1 ↑	*In-vitro* murine GLUTag, *ex-vivo* human colon	(87, 88)
	AR231453, AR435707, AR440006, OEA, 2-0G	PYY ↑, GLP-1 ↑, GI motility ↓	*Ex-vivo* murine gut, *in-vivo* healthy and diabetic mouse, *ex-vivo* human colon	(89, 90)
	Hypergl*+ AR231453	Insulin ↑	*In-vitro* murine MlN6	(88)
	Hypergl. * Compounds A/B^Δ^	Insulin ↑	*Ex-vivo* rat pancreas	(91)
	Hypogl.** Compounds A/B^Δ^	Glucagon ↑	*Ex-vivo* rat pancreas	(91)
	DS-8500a	Insulin ↑, glucagon ↑, GLP-1 ↑, GIP ↑, PYY ↓	Type 2 diabetic humans	(92)
GIPR	Hypogl.**+ GIP	Glucagon ↑	Type 1 diabetic humans	(93)
	Hypergl.*+ GIP	Insulin ↑, somatostatin↑	Healthy humans	(94)
	GIP	IL-6 ↑	*Ex-vivo* human, and murine α-cells	(95)
GLP-IR	GLP-1	Insulin ↑, somatostatin↑, glucagon ↓	*Ex-vivo* healthy murine pancreas	(96)
	GLP-1	Appetite ↓	*In-vivo* intracerebral rat	(97)
	GLP-1	GLP-1 ↑	*In-vitro* murine α-TC 1-6	(98)
	Exendin-4	Glucagon ↓	*Ex-vivo* healthy rat pancreas	(99)
	Exendin-4	Glucagon ↑	*Ex-vivo* diabetic rat pancreas	(99)
TGR5	Hypergl.*+ INT-777, or LCA§	GLP-1 ↑, insulin ↑	*Ex-vivo* healthy human, and murine diabetic islets	(100)
	Taurodeoxycholate	GLP-1 ↑	*Ex-vivo* murine primary ileal cultures	(101)

Analytes are indicated as up (↑) or down (↓) regulated. All in-vivo, or in-human studies, indicate peripheral plasmatic levels.

* (Hypergl.) and

** *(Hypogl.) indicate conditional presence/hyperglycaemia, or absence of glucose/hypoglycaemia*.

§ *(LCA) lithocolic acid*,

† *(INT-777) semisynthetic bile acid, (GSIS) Glucose-stimulated insulin secretion*.

Δ *(Compounds A and B) are experimental GPR119 agonists described by Li et al. (91)*.

A summary of the recognized main activities of all the major GLP-1-secreting receptors, including the GIPR (93, 94), is shown in Table 1.

Many of these chemosensors are also expressed by other enteroendocrine cells, so that the same dietary ligand traveling along the GI tract, leads to the release of multiple hormones.

There are some receptors, such as GPRC6A, with a pleiotropic distribution and still a limited understanding of its physiology. GPRC6A is highly expressed in GLUTag cells, and its activation by L-ornithine has shown to induce GLP-1 secretion (102). Nonetheless, mice deficient for the receptor, show no difference in responsiveness to both L-ornithine and L-arginine (103).

## The physiology of GLP-1

In the last three decades a major tenet seeing GLP1 (7-36)_NH2_, GLP1 (7-37) and the Gastric Insulinotropic Peptide (GIP) as the major contributors of the physiological incretin effect has reached widespread consensus (104). The remaining Glucose-stimulated insulin secretion (GSIS) appears to be enhanced by nutrients, hormones such as CCK, bile acids and endogenous ethanolamides. Animal models show compensatory mechanisms by which, in absence of a major incretin axis, other minor pathways are promoted in the β-cells to maintain their metabolic activity; namely proteins such as GPR119, or the CCK A receptor itself are upregulated, implying a highly plastic metabolic adaptation (105).

Multiple cell types found in the enteroendocrine cell system, the pancreatic islets or the brain have been shown to express the GCG product, a 180 aminoacids long peptide known as proglucagon (PG) (106, 107), which gets trimmed tissue-dependently into at least 6 different bio-active peptides, namely glicentin, oxyntomodulin, glucagon, miniglucagon, GLP-1 and GLP-2 (108, 109). The post-translational processing of the preproglucagon gene into the individual peptides is controlled by two distinct serine proteases, specifically prohormone convertases named Psck1/3 and Psck2, also known as PC1/3, or just PC1, and PC2 respectively (107, 108, 110). PC1/3 and PC2 are responsible for the metabolism of a plethora of peptide pro-hormones, including insulin and GCG among others (111). In particular PC1/3 expressing cells, such as intestinal L-cells and pancreatic β-cells, produce GLP-1, GLP-2 oxyntomodulin and glicentin (110, 112), while PC2 action on PG results in the production of glucagon and its active metabolite mini-glucagon (113, 114). Differential expression of PC genes regulates the hormonal output, and indeed it has been proven that both are expressed along the intestine, with PC1/3 positive cells found more distally than PC2 expressing cells (51), likely secreting glucagon (115). Indeed, the RYGB surgery removes the biggest pool of PC2/glucagon expressing cells from the exposure to nutrients, possibly contributing to the surgical success.

Active GLP-1(7-37), in human and mice is largely metabolized by the enzyme peptidyl-glycine α-amidating monooxygenase (PAM) into the equally active GLP-1(7-36)_NH2_ (49, 116). Both these peptide species are trimmed at their N-term, and inactivated by the ubiquitous protease dipeptidyl-peptidase-IV (DPP-IV), found in the intestinal capillaries, vena porta and liver. Indeed, it has been estimated that just 10-15% of the secreted GLP-1(7-36)_NH2_ reaches the systemic circulation (117), with some authors reporting meager peripheral meal-induced changes in both healthy and diabetic people (118). Furthermore, the DPP-IV product, GLP-1(9-36)_NH2_, is trimmed into GLP-1(28-36)_NH2_ and GLP-1(32-36)_NH2_ by another ubiquitous protease, known as NEP24.11, CD10 or also Neprilysin among other names (119, 120).

Indeed, these once thought inactive metabolites of the recognized GLP-1 receptor agonist GLP-1(7-36) _NH2_ have recently shown to possess multiple beneficial properties. The 9 aminoacids long GLP-1(28-36) protects β-cells from glucolipotoxicity (121), diet-induced steatosis of the liver (122), improves hepatic glucose tolerance in diabetic mice (122–124). Similarly, the 5 aminoacids long GLP-1(32-36)_NH2_ improves glucose disposal, increases energy expenditure and protects β-cells in a diabetic environment *in vivo* (125–127). Indeed GLP-1(9-36) pharmacodynamics studies in human might be partially explained by the activity of its metabolites (128).

These metabolites have possibly important implications for any future treatment of metabolic pathologies such as type 2 diabetes, where our understanding of the pharmacokinetic and pharmacodynamics in humans is virtually absent (128).

In healthy humans, intact GLP-1(7-36) _NH2_ is mainly released by intestinal EECs after the ingestion of food, especially meals rich in fat and proteins (14, 129). Other stimuli, such as physical activity, are also capable to raise its plasmatic levels for up to 90 min after exercise (130).

This hormone generates both short-term and long-term pleiotropic effects. GLP-1 stimulates the β-cells to produce Insulin, blocks pancreatic α-cells' glucagon release via somatostatin (96), slows down gastric emptying (131), improves peripheral glucose tolerance (132), suppresses appetite in the hypothalamus and amygdala (97), increases β-cell mass, GSIS, and elicits protection from glucolipotoxicity (133) and apoptosis (134). Curiously, it also regulates bone physiology (135), and shows anti-inflammatory properties (136).

On the other hand, the most abundant DPP-IV-processed metabolite GLP-1 (9-36)_NH2_, has also been reported to have biological activities, protecting human aortic endothelial cells and cardiomyocytes *in vivo* in dogs (137) and *ex vivo* in mice (138) and rats (139), even in the absence of a GLP-1 Receptor (140, 139). Some authors postulate the existance of an unknown GLP-1(9-36)_NH2_ receptor (141, 142), because indeed this cleaved peptide is found in peripheral blood at one order of magnitude higher concentrations than “active” GLP-1 (7-36)_NH2_ and shows cardioprotection, antioxidant properties (138) and appears capable to also inhibit hepatic neoglucogenesis (141).

GLP-1 (7-36)_NH2_ itself is known to have general protective and modulating cardiovascular effects (143), as shown by different commercial GLP-1 mimics with proven cardioprotection type 2 diabetes (144).

In healthy fasted individuals, it is recognized that peripheral plasmatic active GLP-1 (7-36)_NH2_ plasmatic levels hover around 5 pM, but within 5–10 min after an oral glucose load, they start to rise, up to a maximum of less than 10 pM after 40–90 min, and slowly descend back to baseline values in 150 min. On the other hand, the cleaved GLP-1 (9-36)_NH2_ summed to the GLP-1 (7-36)_NH2_ to give what is normally referred to as total GLP-1 levels, raise up to more than 40–60 pM (108). In perspective, GIP and Insulin show much broader dynamic ranges, with post meal levels reaching 300 and 400 pM respectively, from their baselines <20 pM within 30 min post glucose ingestion (145, 108). Curiously, some bariatric RYGB patients experience up to a 10-fold increase in post-meal active GLP-1 plasmatic levels (from fasting 5 pM to post-prandial 30–65 pM) (146), and have a 2- to 3-fold higher glucose-stimulated Insulin secretion (147), which in some diabetic patients results in GLP-1-mediated hyperinsulinemic hypoglycaemia that requires GLP-1 antagonism or surgical reversal of the intestinal anatomy (148).

Different authors consider the success of surgical intervention a consequence of a major change in gut hormonal profile, primarily a supra physiological post-prandial GLP-1 secretion (29, 30). This reasoning fits with the observation that type 2 diabetic patients display a shorter post-prandial peak of GLP-1, hence they are deficient for the longer response seen in healthy individuals. Multiple groups describe diabetic patients with lower plasmatic GLP-1 but heightened GIP levels and β-cell resistance to the stimulatory effect of both GLP-1 and GIP (18, 149–153,).

Nonetheless, different animal models deficient for GLP-1 signaling, in addition to human studies, prove the dispensability of GLP-1 for surgical success (31–34), questioning the causative nature of GLP-1 for the reported metabolic benefits.

On the other hand, PYY has been proven to be upregulated, and necessary, for RYGB mediated restoration of the diabetic islets, and overall cure of diabetes in rats (35) and humans (154).

Another important source of endogenous GLP-1 is the brain, a tissue where it acts as a neurotransmitter. Indeed central GLP-1 production appears essential, since peripheral GLP-1 is assumed to not be able to cross the blood-brain barrier (BBB). In particular, neurons of the hindbrain found in the nucleus-tractus solitarius (NTS) secrete GLP-1 and activate hypothalamic neurons of the paraventricular nucleus (PVN), resulting in satiety (155, 156). Indeed it is clear that PC1/3 dominant neurons of the NTS express also other the PG peptides oxyntomodulin, glicentin, and GLP-2 together with GLP-1 (157). Although expressed at much lower levels, PC2 activity has also been recognized in these neurons, and traces amounts of glucagon might have important implications.

NTS neurons-derived GLP-1 appears to reach out to multiple locations within the central nervous system (CNS), which have been proven to express the receptor, and be activated after a central administration of GLP-1 receptor agonists. These areas include the NTS itself, the supraoptic nuclei, the arcuate nucleus (ARC) and the area postrema (AP) other than corticotropin-releasing hormone (CRH) PVN neurons (158, 159). Beyond satiety, this signaling appears to be a key factor for neuroprotection (160) insulin sensitivity and glucose metabolism (158).

Curiously, the feeling of satiety, is also achieved by another neurotransmitter, the Cocaine- and amphetamine-regulated transcript (CART) (161). This peptide, acts also as a hormone, and is expressed by both β-cells and intestinal GLP-1 and GIP producing cells causing GLP-1 secretion *in vivo* via a yet unknown GPCR (162).

It is not entirely clear to what extent endogenous GLP-1 activates all the reported GLP-1 receptor expressing neurons and to what extent it depends on the CART peptide especially in type 2 diabetes or obesity. Nonetheless, some commercial mimics of GLP-1, such as Liraglutide, even when administered peripherally, appear to cross the BBB and activate neurons within the ARC resulting in GABA dependent inhibition of neuropeptide Y (NPY) and agouti-related peptide (AgRP) secretion. This signaling has proven to be essential for the Liraglutide mediated weight loss in rats (163). GLP-1R expressing hypothalamic neurons have proven dispensable for the beneficial metabolic activity of both BBB permeable Liraglutide and Exending-4 (164).

Singularly, BBB impermeable mimics of GLP-1 have still shown to activate GLP-1 Receptor expressing neurons (165), but they require a functional gut-brain axis through the vagus nerve (166). In particular, vagal afferent neurons expressing the GLP-1R are necessary for GLP-1 mediated induction of satiety (167) but not glucose lowering effects (168).

The complex inter-organ pharmacokinetic of GLP-1, compounds into a convoluted pharmacodynamics encompassing multiple metabolic systems.

Indeed the GLP-1(7-36) _NH2_ receptor, a GPCR, is found to be expressed by a wide range of tissues and cells such as: α, β, and δ-cells (169), sinoatrial node myocytes, arterial smooth muscle cells of lungs and kidneys, megakaryocytes, macrophages, monocytes, lymphocytes, gastrointestinal tract mucosa [mainly Brunner's gland in the duodenum, but also in the parietal cells of the stomach, jejunum ileum and the nerve plexus around the small and large intestine (170, 171)], central nervous system [neocortex, cerebellum, thalamus, amygdala, area postrema, hypothalamus, hippocampus, nucleus tractus solitarius (158)], peripheral nervous system (myenteric plexus) and in the skin (14, 172–176).

Counterintuitively, mice completely defective for the GLP-1 receptor were reported to be protected from high-fat diet-induced peripheral Insulin resistance (177) and, consistently with this, central inhibition of GLP-1R signaling with the antagonist exendin 9-39 improves glucose tolerance and glycaemia (178). Conversely, mice defective for both the receptors for glucagon and GLP-1, or GLP-1 and GIP, show a highly plastic entero-pancreatic system that adapts and gives these animals no overt phenotype in terms of glucose homeostasis (105).

Nonetheless, the pharmacological activation of the GLP-1R is clinically beneficial (179), offering an improved glycaemic control with lower cardiovascular morbidity and without the risk of hypoglycaemia associated with some current anti diabetic drugs (173). Furthermore, being an appetite suppressant, GLP-1 signaling also helps to lose body weight, especially if in combination with metformin. Conversely, anti-diabetic drugs such as sulfonylureas, or Insulin, are known to induce not only weight gain (180, 181), but also an increased risk of hypoglycaemic events (182). Pharmacological activation of the GLP-1 Receptor has also shown to help exogenous insulin in the control of glycaemia in patients with type 1 diabetes, by slowing the gastric emptying and blocking glucagon secretion (183, 184).

Currently, six different peptide GLP1-Receptor agonists are on the market, with more in clinical trials. In particular, two short-acting formulations of Lixisenatide and Exenatide and four long acting preparations of Exenatide, Liraglutide, Dulaglutide and the most recent and successful Semaglutide, were approved in October, 2017 for the North American markets by FDA^1^ (25, 185). The first GLP-1 analog to be approved by FDA in 2005 for the management of Type 2 diabetes was the chemically synthesized Exenatide under the name of Byetta (186), a formulation of the DPP-IV resistant peptide discovered in the gila monster *Heloderma suspectum* saliva in 1992 (187). Despite the longer half-life in serum, Byetta needs to be injected twice a day. In the last decade, formulations with extended release entered the market with once-weekly self-administrations pens.

Pleiotropic beneficial effects have been reported for this class of drugs. Beyond the improved glycaemia control, essential for the short term treatment of diabetes (188), different GLP-1RAs are powerful clinical tools for the management of diabetic kidney disease (DKD) (28, 189) non-alcoholic steatohepatitis (NASH) (190), neuroinflammation (191), obesity and cardiovascular disease (192–195).

Although GLP-1RA are improving the lives of patients affected by type 2 diabetes or the metabolic syndrome (196), the physiology of GLP-1 is far from being clear.

More recent data suggest how the unimolecular co-activation of GLP-1 and GIP receptors, has powerful anti-diabetic effects superior to either agonism (197). Furthermore, oxyntomodulin is a natural dual-agonist of GLP-1 and glucagon receptors and displays anti-diabetic properties in humans (198, 199). Upon this finding, a tri-agonist peptide, targeting the receptors of GLP-1, GIP, and glucagon was created (200). The *in vivo* effects of this drug are unparalleled, even superior to what can be achieved with the dual agonists for either combination. The synergistic activation of these three important receptors is capable to revert diet-induced obesity, cognitive impairment and T2D in mice models, warranting future human studies (201, 202).

## Expanding the physiology of GLP-1

**Figure 2:**
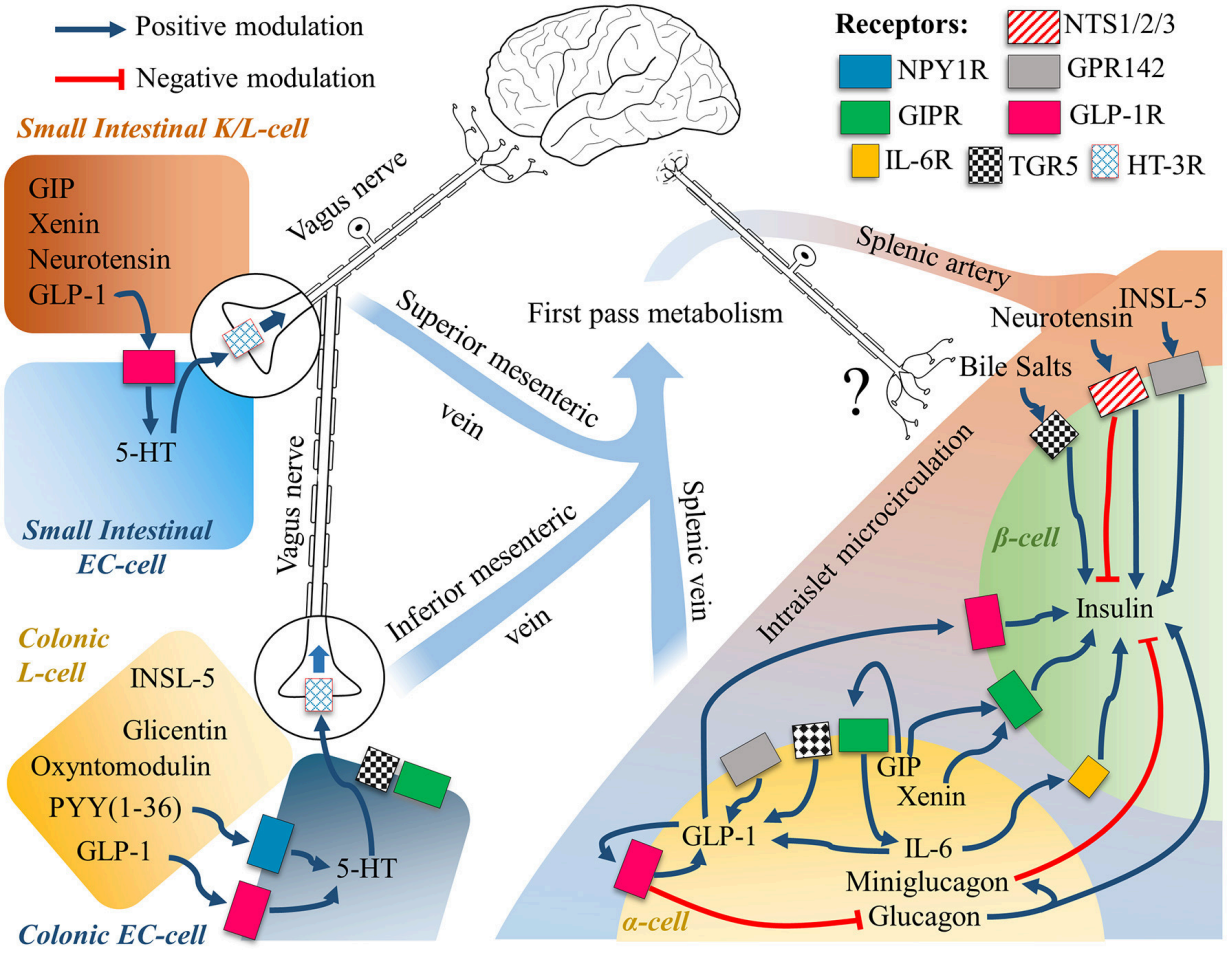
The gut-brain-islet axes of GLP-1. The intestinal EECs secretome is subject to first pass metabolism, while intraislet signaling relies on paracrine signaling. Intestinal cells are known to communicate with the Enteric Nervous System, and the Central Nervous System through the Vagus Nerve. Neuronal engagement between the gut lumen and the islets of Langerhans is a possible compounding explanation to the incretin effect, whereby the mechanistic of the single molecular players are still largely unknown. See text for further details.

When examining the physiology of glucagon-like peptide-1, it is important to consider that there is an expanding body of evidence that questions its systemic endocrine physiology (203, 204). Pancreatic α-cells have been demonstrated to express and secrete not only GLP-1 (205, 206), but also PYY (35) GIP (207, 208) mini-glucagon (209) or even Xenin (210) together with glucagon (Figure 2). The key protease responsible for the processing of the proglucagon peptide into GLP-1 is Psck1/3, which has shown to be upregulated in α-cells during hyperglycaemic, hyperlipidemic, or inflammatory conditions to promote glucose-induced glucagon suppression, a compensatory response to a metabolic insult as in type 2 diabetes (205). Insulin itself has shown to modulate PC1/3 expression to possibly aid its own metabolic activity (211).

Recently, the whole dogma of the role of intestinal GLP-1, envisioning the traveling from the gut to the liver and ultimately reaching the pancreatic β-cells to bind its GLP-1R has been questioned in transgenic mice (204). Indeed, since both DPP-IV degrades and NEP24.11 degrade GLP-1 within seconds, the possibilities of any intestinal GLP-1 to reach the system circulation and then the islet microcirculation are doubted. Besides, it is important to consider that intestinal GLP-1 has a local concentration in the nM range (10–100 pico moles per gram of tissue, see Figure 1), further advocating that the main action of this protein have evolved to be locally restricted.

Animals deficient for the GCG gene in the intestine, still experience a normal incretin effect disrupted with the GLP-1R antagonist Exendin (9-39) (204). This indicates that it is the intra islet, α-cell derived GLP-1 that shows the meal-induced insulinotropic properties. A critic to the use of a murine model deficient for intestinal GCG products, would be that other gut hormones might compensate for the lack of a functional GCG gene in that tissue, hence explaining the normalized incretin effect. Indeed other gut hormones such as GIP must be responsible for the incretin effect to a higher degree than once thought. Nonetheless, it is also clear that intra-islet GLP-1R signaling is essential for GSIS, with more evidence that an intra-islet paracrine GLP-1 signaling is physiologically present (212, 213) and necessary for β-cell health under metabolic (214).

In contrast, mice deficient for GLP-1R only in β-cells have a normal incretin response and oral glucose tolerance, indicating the dispensability of intra-islet signaling of GLP-1 for the incretin effect. Interestingly, these same animals have an improvement of their glucose tolerance in response to oral DPP-IV treatment, but not to subcutaneous GLP-1 mimics, indicating how the former relies completely on localized, non β-cell GLP-1R (215).

There are still multiple gaps into our understanding of how different GLP-1 producing tissues communicate, especially in the brain to islet axis. It is known that acute, but not chronic, central GLP-1 receptor activation directly modulates glucose-induced Insulin secretion implicating a direct brain to islet neuronal communication (61).

On the other hand, chronic GLP-1 activity in α-cells increases its own secretion, feeding an autocrine loop that gets overstimulated with the use of exogenous synthetic GLP-1R agonists [(98); Figure 2]. Curiously in diabetic rats, it has recently been shown that this loop might indeed induce the production of more glucagon than in healthy animals (99).

It has been known for more than two decades and has been confirmed more recently, that an infusion of GLP-1(7-36)_NH2_ has insulinotropic and glucagonostatic effects. This is seen when the plasmatic levels are above 50–60 pM, equivalent to more than five times the levels seen post-prandial in healthy individuals challenged with a bolus of glucose, or 10-fold their basal levels (153, 216), adding further doubt to the physiological hormonal dogma of intestinal GLP-1. Considering the mounting evidence, it is clear that we need to understand what hormonal and/or neuronal signals are bridging the gut luminal content to the insulin secretion explaining the incretin effect. Given that Intestinal oxyntomodulin, glicentin, glucagon and GLP-1 expression have proven to be dispensable in mice (204); other intestinal hormones such as GIP, PYY, Neurotensin, INSL-5 or the GIP co-secreted Xenin (217) might play an important role (Figure 2). Currently, not much is known about the physiology of Neurotensin, INSL-5 and Xenin. The first two have been reported to be co-expressed with GLP-1 in the small and large intestine respectively, with Neurotensin being reported also in pancreatic β-cells (210), while Xenin in a sub population of duodenal GIP positive cells and α-cells. Neurotensin levels are correlated with leptin (218), rise in response to fatty meals, signals through two different G-protein coupled receptors known as NTSR1 and 2, and a third single transmembrane receptor, NTSR3, also known as sortilin (219). All of these receptors are expressed by pancreatic β cells, where their activation appears to mediate insulin release at low glucose levels and blockage at high levels (219, 220), (see right side of Figure 2). On the other hand, INSL-5 targets a GPCR known as GPR142, also known as RXFP4, a receptor found to be expressed by the NCI-H716 cell line (54), and both α and β-cells in the pancreas, and its activation directly stimulates the expression of GLP-1 and insulin, representing a possible new pharmacological tool for the treatment of type 2 diabetes (77, 221), and supporting a possible role for INSL-5 in the incretin effect. Xenin is another gut-derived food-induced peptide known to potentiate GIP activity (222, 223). Considering that α and β-cells express GIPR (224) and that the GIP-potentiating activity of Xenin has been reported to be lost in human diabetics (223), it appears to be a critical player in this disease, likely involving the activity of GLP-1.

In addition, both *in vitro* and *in vivo* Interleukin-6 (IL-6) has shown to be a powerful GLP-1 secretagogue, capable to positively modulate both the proglucagon gene, and the expression of PC1/3 in α-cells and intestinal L-cells (225, 226). Indeed, GIP has shown to not only be co-expressed with GLP-1 and glucagon in α-cells (207); it also stimulates in an autocrine/paracrine fashion the expression of IL-6 in the same α-cells, thus indirectly acting as a GLP-1 secretagogue (95).

IL-6 has shown also to induce the secretion of intestinal GLP-1, indirectly via the release of adipocytes derived Leptin (227).

Curiously, it was recently reported that this pro-inflammatory cytokine, IL-6, similarly, but independently from GLP-1, slows gastric emptying (228). Furthermore an inflammatory status, as seen in pathologies such as type 2 diabetes, might compromise the gut mucosal permeability, leading to the exposure of intestinal EECs to luminal LPS, and a TLR4-mediated release of GLP-1 (229). This is consistent with the knowledge that GLP-1, as well as glucagon, has shown to possess powerful anti-inflammatory properties *in vivo*, an area that hold with vast therapeutical potential (136, 230).

Ghrelin is another possible player, since it has been proven to be expressed not only in the gut, but also in a distinct subpopulation of islet cells named ε-cells (231) and, being known to be a stress-induced (232) GLP-1 secretagogue (233, 234), it might play an important role in the intra-islet signaling.

Recently, it has been demonstrated that mice with a deletion of the GLP-1 receptor only in β-cells, are resistant to the beneficial anti-diabetic effect of a vertical sleeve-gastrectomy (36), suggesting how GLP-1 activity in β-cells is key to the bariatric surgery success. It is not known if intra-islet α-cells production of GLP-1 is affected by the surgical procedure or, more importantly, how this axis is impaired in the metabolic syndrome, type 2 diabetes and related pathologies.

It appears that only in RYGB and SG patients intestinal derived GLP-1 has a true endocrine role, while in healthy individuals, localized, paracrine and neuronal signals primarily define the GLP-1 physiology.

It is therefore clear that currently available GLP-1RAs, mimicking on the peripheral action of GLP-1 (7-36)_NH2_, not only ignore the yet unknown physiology of GLP-1 (9-36)_NH2_ or its metabolites, but they also fail to address the tissue specific physiology of GLP-1 (7-36)_NH2_, while pushing to supra-physiological limits the endocrine GLP-1 receptor axis, likely explaining the reported side-effects and only partial success in the treatment of T2D.

In addition, it is important to notice that the ubiquitous DPP-IV protease targets not only GLP-1 but also oxyntomodulin, GIP and PYY among other proteins (235). Specifically, the GLP-1 co-secreted cousin PYY(1-36), agonist of the vasoconstrictive Y(1) receptor, is physiologically trimmed by DPP-IV to give rise to the appetite-suppressant, anti-diabetic and blood-brain barrier permeable PYY(3-36) agonist of Y(2) receptor (220). It is therefore clear that pharmacological DPP-IV blockage disrupts this axis and induces hypertension (236).

Recent studies provide new evidence supporting the paracrine nature of intestinal GLP-1, whereby Serotonin-(5-HT)-secreting enterochromaffin (EC) cells are directly stimulated by locally produced GLP-1, which in turn stimulate afferent Vagal nerves (Figure 2) bridging the gut to brain axis. Accumulating evidence suggest that, especially in the colon, EC cells express multiple receptors for the microbiome metabolites, representing a new important link bridging the microbiome to the brain (237, 238).

A better way to amend the pathophysiology of GLP-1 reported in diabetes or other diseases, would be to induce tissue specific *de novo* GLP-1 production, leading to a more physiological and likely safer, short and medium distance signaling. Numerous attempts have been made with multiple GLP-1 secretagogues such as GPR119 agonists (239) but so far no compound has reached the market because of bioavailability issues and systemic off-target toxicity. One possible way to minimize the side-effects of the single drugs is to combine them to achieve synergistic effects, as reported recently with a combination of a DPP-IV inhibition, SSTR5 antagonism and GPR40 and TGR5 agonism, capable to raise circulatory active GLP-1(7-36)_NH2_ levels to more than 300-400 pM in mice (240).

## Sweetness in the gut

Studies *in vitro* and *ex-vivo* with isolated human primary cells suggest that there are two temporally distinct pathways that lead to the glucose-stimulated release of GLP-1, similarly to what happens in β-cells with the 1st or 2nd phase insulin release. A quick mechanism independent of the cell energetical state and a slower one, metabolism dependent, mediate the release of this incretin (53, 72).

The 1st phase in the pathway of glucose signaling, sees the electrogenic sodium-coupled glucose transporters 1 (SGLT1) mediated uptake of two Na^+^ ions for every internalized glucose molecule (53). This depolarization is propagated through voltage-dependent Calcium and Sodium channels, which currents lead to the discharge of the hormones containing vesicles (72).

The 2nd phase is exemplified by the absorption of simple sugars, such as Glucose or Fructose, via the facilitative transporters GLUT2 and GLUT5 respectively, which leads to an increased internal metabolism mirrored by intracellular ATP levels. This state leads to the blockage of ATP dependent potassium channels and the subsequent membrane depolarization, followed by the secretion of the hormonal cargo.

Mace et al. (241) demonstrated how diazoxide, a K^+^ATP channel opener, completely abolished the glucose-dependent incretin release while a channel blocker, tolbutamide, exacerbates it in terms of secreted GLP-1, GIP and PYY.

More recent data, question the first mechanism in enteroendocrine cells. Glucose mediated GLP-1 release happens in humans only in the proximal and distal small intestine and independently of ATP mediated potassium channels closure. Furthermore, concentrations of up to 300 mM glucose do not induce GLP-1 secretion from colonic human mucosa because GLP-1 producing L-cells barely express SGLT1 (43, 53, 58, 72).

Consistently, the use of α-methyl-D-glucopyranoside (MDG), an acaloric substrate of SGLT1, within 5 min triggers the release of GLP-1 as glucose does, demonstrating how it is the sodium current that triggers the release of the incretin, and not the metabolic ATP-driven arrest of potassium currents and following calcium spike (58).

The pharmacological blockage of SGLT-1 with phloridzin, in a rat small intestine perfused system, results in just a halved secretion of GIP, GLP-1, or PYY, and the addition of phloretin, a GLUT2 inhibitor, brings these values down to basal levels. In fact, this double blockage of SGLT1 and GLUT2, completely inhibits the responsiveness to other stimulants as well, such as sucralose, glycylsarcosine, OEA, propionate and taurocholate. The activity of the calcium channel CasR is also essential for the responsiveness to free aminoacids (241).

All these observations are challenged by longer term *in vivo* studies. Blockage of SGLT-1 markedly improves glucose-stimulated GLP-1 release if a 3-h long period is considered.

The rationale given by Oguma et al. (242) is that SGLT-1 is expressed mainly in the small intestine, hence its inactivation results in heightened luminal glucose that travels down to the colon where it someway stimulates GLP-1 release. Given the fact that SGLT-1 is barely detectable in colonic proglucagon positive cells and that potassium channels in this tissue are unresponsive to sulfonylureas, the molecular sensor(s) that causes the release of GLP-1 *in vivo*, remains elusive.

Another enigmatic G protein is α-gustducin, a key element in sweet-taste transduction pathways downstream of the heterodimer formed between the GPCRs Tas1R2 (T1R2) and Tas1R3 (T1R3).

Its expression has been reported in colonic L-cells and appears to be responsible for the glucose-stimulated release of incretins (243, 244). This is confirmed by the impaired glucose-stimulated release of GLP-1 in mice lacking either T1R3 or α-gustducin (244).

Interestingly, this axis is also activated by the disaccharide sucrose and by the non-metabolizable and therefore anergic sucralose (243). Of note also Aspartame, Acesulfame K, Glycyrrhizin and Saccharin bind the sweet receptor heterodimer Tas1R2/3 and they have shown to stimulate GLP-1 secretion in the human duodenal adenocarcinoma-derived HuTu-80 cell line (245, 246). Despite this report, other groups weren't able to replicate these results (53). Indeed, it was shown that proglucagon expressing cells, derived from the colon of Venus mice cultures, were not responding significantly to Sucralose (1 mM) in terms of both released GLP-1 and intracellular Calcium. Conversely, proglucagon negative cells responded to the sweetener. More doubts about the role of Tas1 receptors were raised after the demonstration that oral gavage with sucralose, saccharin, stevia, acesulfame potassium or tryptophan do not cause a gut incretin release in Zucker diabetic fatty rats (247).

## Long and middle chain fatty acid receptors

The study of the receptome of enteroendocrine cells, has provided invaluable pharmacological insight with the discovery of proteins capable to sense multiple compounds once thought to be only nutrients.

A prime example is given by two GPCRs, GPR40 and GPR120, also known as Free Fatty Acid Receptor 1 (FFAR1) and 4 (FFAR4) respectively. These chemosensors are two major molecular players in the detection of dietary, medium (C8-12) and long (C14-22) chain fatty acids (LCFA) (84, 248,).

GPR40 is primarily expressed by the pancreatic β-cells, where it plays a pivotal role in FFA-mediated insulin secretion (249) but also in α-cells (78, 250), CCK (80), GIP (251), and GLP-1 (79) producing cells in the gut and in hypothalamic neurons (248, 252, 253). Animals deficient for this receptor are protected from obesity-induced hepatic steatosis, hyperinsulinemia, hypertriglyceridemia and hyperglycaemia. More than a decade ago a study showed that GPR40 mediates the long-term FFA-induced lipotoxicity seen in the diabetic islets (254); nonetheless, these findings are still under debate today. Recent data are still highly polarized, with some authors supporting (255), and others disproving this (256), or even indicating that GPR40 protects β-cells from lipotoxicity (257) rendering difficult to draw any conclusive mechanistic involvement in healthy and diabetic individuals. Nonetheless, the activation of this receptor with FFAs has demonstrated to induce the secretion of incretins (79, 258) glucagon (78, 250) and partially glucose-stimulated insulin (259, 260) reducing food intake, and lowering body weight in animals models (261). Mice without a functional GPR40 display an impaired CCK and GLP-1 secretion after an oil gavage, while surprisingly animals deficient for GPR120 display a normal corn oil-induced GLP-1 secretion (80, 262).

GPR40 is coupled to both Gq and Gs proteins and *in vivo* studies suggest how signaling through both these cascades elicits the most powerful GLP-1 secretion (258). Ligands that bind GPR40 and activate predominantly only the Gq pathway are not good GLP-1 secretagogues. Indeed recently it has been shown that dietary triglycerides appear to induce the secretion of GLP-1 via GPR40 in synergy with the Gs activating GPR119 (263). Nonetheless, chylomicrons have been reported to be powerful GPR40-Gq activators and GLP-1 secretagogues, acting from the basolateral side of the intestinal mucosa (264).

The two synthetic GPR40-specific compounds AM-1638 and AM-5262, have been found to act as double Gq and Gs agonists but also as positive allosteric modulators, capable to enhance the GLP-1-secreting capabilities of Gq-only agonists such as dietary docosahexaenoic (DHA) and α-linolenic acid (ALA), independently of the orthosteric site (265).

**Figure 3:**
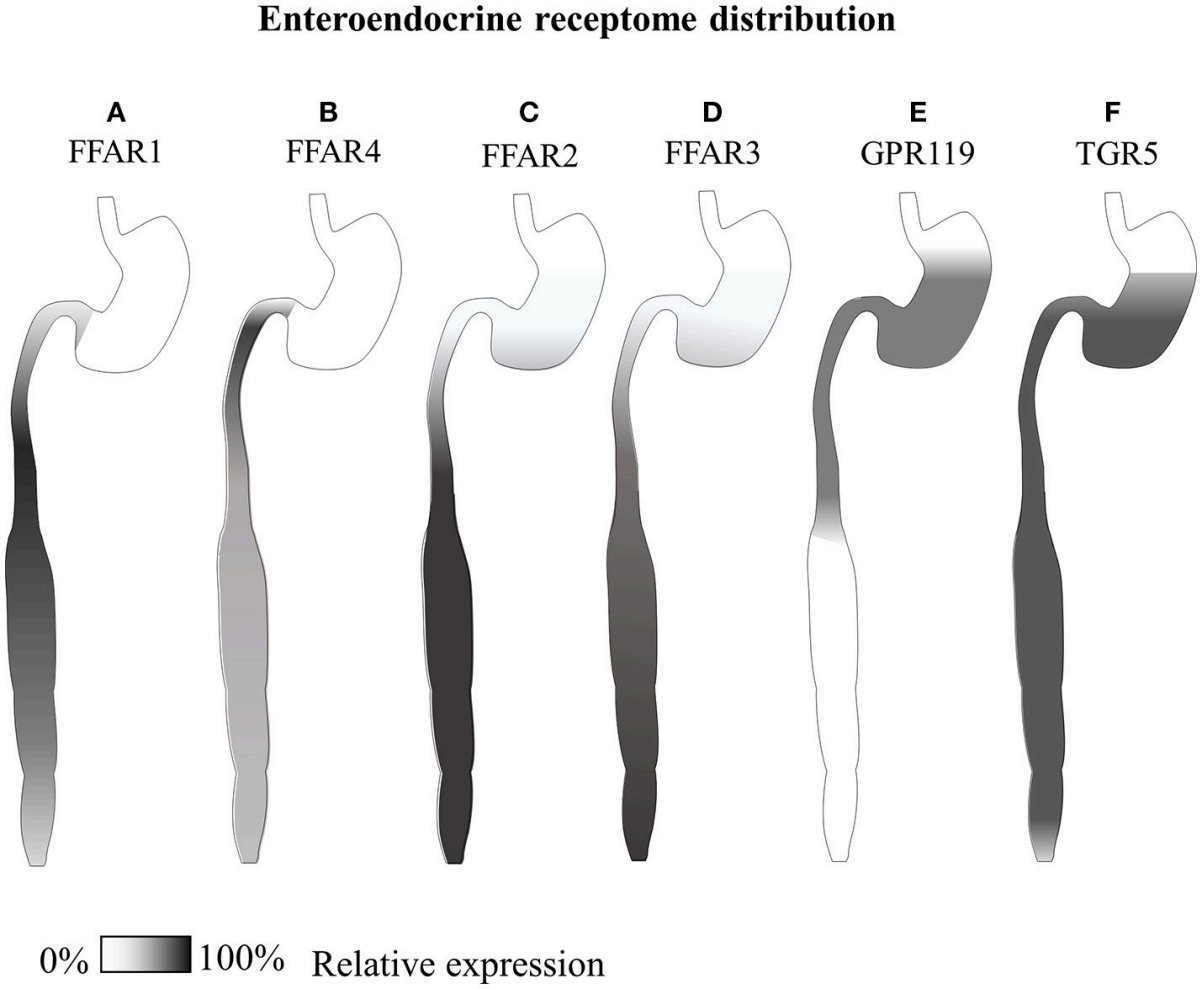
Gastrointestinal GLP-1-secreting receptome distribution. Summary of available expression studies of different GLP-1-secreting receptors along the gastrointestinal tract. **(A)** GPR40/FFAR1 has been reported to be expressed in the small intestine in different EECs, with overall higher transcript levels than GPR120, and superior co-localization with GIP In the distal small intestine (79, 80, 266). **(B)** GPR120/FFAR4 has shown co-expression with both proximal small intestinal GIP^+^ and large intestinal GLP-1^+^ cells (85, 266–268) **(C)** GPR43/FFAR2 and **(D)** GPR41/FFAR3 are co-expressed by all types of enteroendocrine cells, from the stomach to the rectum, especially in the colon (83, 269, 270). **(E)** Reports of comparative GPR119 transcript are contradictory, while immunohistochemical data indicate co-localization with a minor fraction of CCK and GLP-1 positive cells mainly in the stomach and small intestine (271, 266). **(F)** TGR5, has been reported equally distributed along the whole gastrointestinal tract of dogs (101, 272, 273).

GPR120 shows very little sequence similarities to the other free fatty acid receptors but, likewise, is found to be expressed by the enteroendocrine cell system, especially in the colon (see Figure 3), but also in the lungs (267), white and brown adipose tissue (274, 275), hypothalamic microglia (253), macrophages and, contrarily to GPR40, not in β-cells but in somatostatin producing δ-cells (276). Both small intestinal GIP and colonic GLP-1 secreting cells express GPR120, and the molecular cascade triggered by this receptor has been shown to mediate dietary incretin release directly or indirectly through CCK (84–86). Interestingly, both of these two receptors are expressed only by a fraction of hormone positive EECs; in particular, it has been reported that only 3% of GLP-1 positive cells express GPR40, and 23% GPR120 (266).

GPR120 displays a ligand preference similar to GPR40; a broad range of long chain fatty acids signal through it, with some ligands eliciting more robust calcium responses than others (84). Multiple dietary compounds have shown to be powerful agonists of GPR120, such as pinolenic acid, a poly-unsaturated fatty acid (C18:3 *trans, cis, cis* Δ 5, 9, 12) found in pine nut oil (277), or the yeast derived phytosphingosine (278).

In macrophages and adipose tissue, GPR120 mediates ω-3-mediated anti-inflammatory and insulin sensitizing effects (279, 280). Contrarily to GPR40, the genetic deficiency of GPR120 is more dramatic. Knockout animals show hyperinsulinemia and insulin resistance, hyperglycaemia and osteoarthritis (281), hepatic steatosis and therefore obesity. Furthermore, an absence of GPR120, results in an overactive glucagon signaling, explaining the hyperglycaemia (282). Indeed, in humans, a single aminoacid mutation of the receptor that hampers its signaling is associated with obesity and insulin resistance (283). Expectedly, GPR120 agonism shows powerful anti-diabetic, anorexic, and hepatoprotective properties in multiple animal models (275, 284–287), at least partially mediated by GLP-1 (288).

Considering the overlap of natural ligands of GPR40 and GPR120, it has been difficult to study them individually and understand their individual physiology, while recent data indicate that indeed these two receptors work synergistically, to exert anti-diabetic activity *in vivo* from the gut (289), and the brain (253).

Despite these advancements, in clinics there are currently no available drugs targeting GPR40 and GPR120. TAK-875, the best candidate for GPR40 which showed promising GSIS capabilities up to Clinical Phase III for the treatment of T2D, had to be halted because of hepatotoxicity and alteration of bile salts composition (290).

Despite these setbacks, encouraging animal data warrant future efforts for the development of new drugs capable to activate synergistically both GPR40 and GPR120 and mediate, through GLP-1 and other intestinal, pancreatic and cerebral peptides, better treatments for multifactorial chronic metabolic diseases.

## Short chain fatty acid receptors

In 1997, four 7 α-helixes transmembrane receptors, GPR 40, 41, 42, and 43 were mapped on the same locus found on the long arm of chromosome 19 (291). Soon after, different groups identified GPR 43 and 41 as the receptors for free fatty acids, which were then chronologically renamed FFAR2 and FFAR3 respectively (292–294).

Both these receptors are activated by similar types of short chain fatty acids (292), and both these signal through an inhibitory G type protein, but FFAR2 is also capable to signal through Gq/11 proteins (293) by which it has shown to mediate GLP-1 and PYY secretion *in vitro* and *in vivo* (82, 295).

Along the gastrointestinal tract, both GPR 41 and 43 have been reported to be co-expressed, with FFAR2/GPR43 at higher levels and overall number of cells, especially intraepithelial leukocytes, while FFAR3/GPR42 is found on submucosal neurons [see Figure 3, (83, 295–297)]. Indeed FFAR2 holds promise for the management of Inflammatory Bowel Disease (IBD) (298) a possible side-effect of anti-diabetic treatment with DPP-IV inhibitors (299).

Feeding rats with fructo-oligosaccharide as a source of SCFAs has also shown to upregulate FFAR2 (270). Recently, both the receptors have shown to heteromerize *in vitro*, eliciting synergistic signaling and β-arrestin-2 recruitment (300). Furthermore FFAR2 activation *in vivo* with an inulin-enriched diet in mice results in PYY release and proliferation of L cells *in vitro* (81). Nonetheless, there is still some controversy on the *in vivo* involvement of FFAR2 and FFAR3 in GLP-1 modulation (301, 302), with some reports indicating that blockade of GPR43 *in vitro* releases GLP-1 (303) and others indicating different mechanisms of action, with FFAR2 releasing PYY from intestinal L-cells (81), while FFAR3 restricted to submucosal neuronal activity (295) despite its apparent expression by the majority of enteroendocrine cells (83).

In pancreatic β-cells, both GPR43 and GPR41 are expressed, and the latter antagonizes GSIS (304).

Adding complexity to the study of these receptors, there is extensive species-specificity, so that animal findings result in poorly translatable data, requiring the generation of complex human-murine chimera currently under intense study (305, 306).

Nonetheless, considering that the half-maximal effective concentration (EC_50_) for Acetate, Propionate, and Butyrate is around 0.5 millimolar upon both GPR41 and GPR43 (292) and that the SCFA concentration in the human ileum and colon lumen is superior to 100 millimoles per kg (307–309), it is likely that both receptors are constitutively active. Obese patients, have been reported to produce more SCFAs in their intestines (310), but indeed meaningful diet-induced shifts in SCFA production fluxes have proven not sufficient to modulate peripheral levels of GLP-1 and PYY (311).

GPR42 is another G-Protein-Coupled-Receptor that was initially considered to be an inactive pseudogene derived from GPR41. In 2009, 29% of 202 human alleles of GPR42 were shown to have an inactivating single nucleotide polymorphism (SNP) at W174, and 61% with an arginine in like GPR41, resulting in a fully functional receptor, differing from it by only 5 aminoacids (312). A more recent study highlights how GPR42 is not only functional, but displays a pool of haplotypes in a great proportion of humans, with a distinct pharmacology (313).

## GPR119

GPR119, also known among other names as glucose-dependent insulinotropic receptor (GDIR), was independently discovered less than two decades ago by several groups around the world and deorphanized soon after with the discovery of Oleoylethanolamide (OEA) as its first endogenous ligand (314–316).

Recently our group has demonstrated that indeed OEA is just a partial agonist of GPR119, and the biological ligand of this receptor is the lysophospholipid Oleoyl-Lysophosphatidylinositol (Oleoyl-LPI) (87). This bioactive lipid induces a powerful GPR119 mediated-GLP-1 secretion *in vitro* and *ex-vivo* from intestines of wild type, but not GPR119 deficient mice. This peculiarity is not shared by LPI species with different aliphatic chains, which have been described as the ligands of GPR55 (317).

This GPCR is primarily expressed in the pancreas by α-cells, β-cells and γ-cells (271, 318, 319), and is found at lower concentrations along the GI tract, especially in the stomach and duodenum, where counterintuitively only a minor fraction of CCK, and GLP-1 expressing duodenal enteroendocrine cells display GPR119 (266, 271). This receptor is also expressed, and hence can be studied, *in vitro*, by the human enteroendocrine cell model NCI-H716 or by the murine GLUTag cell line (320). Heterologous expression *in vitro* unveiled its constitutive activity capable to raise intracellular cAMP levels through Gαs (321) and lead to the secretion of GLP-1 and PYY (89). Rodents, contrarily to humans, express GPR119 also in some regions of the brain (316). The activation of this receptor is known to mediate glucose-stimulated insulin secretion and a glucose-independent release of incretin hormones by intestinal enteroendocrine cells (88).

Long-chain fatty acids and phospholipids like lysophosphatidylcholine (LPC), other compounds such as retinoic acid (RA) and multiple N-acylethanolamines (NAE) such as N-oleyldopamine (OLDA), palmitoylethanolamide (PEA), or oleylethanolamide (OEA), all act as endogenous ligands of GPR119. OEA is a more potent GPR119 agonist than its glycerol ester 2-Oleoyl Glycerol (2-OG) found in olive oil (322).

Indeed, oleic acid is internalized via CD36 and converted to OEA in the duodeno-jejunal enterocytes, which in turn causes satiety directly via PPAR-α (323) or indirectly through an incretin secretion mediated via GPR119 in the gut (324). Curiously, fat-induced OEA synthesis is a fairly conserved pathway in metazoan, being present in fish and extremely slow-metabolism reptiles such as pythons (325, 326).

Triglycerides, with medium length fatty acids such as 1,3 Dioctanoyl- 2 Oleoyl-glycerol, can also cause the release of GLP-1 in humans. However, this happens via the metabolized 2-OG component, since dietary medium chain fatty acid do not cause any appreciable release of incretins (322).

Counterintuitively, long term olive oil feeding does not improve glucose tolerance or insulin responses in diabetic rats (5). Indeed, more recently it has been reported that a high-fat diet enriched in oleic acid leads to an impaired endogenous OEA and other N-acylethanolamides intestinal production in mice (327), suggesting that a chronically resistance is taking place within the OEA synthesis pathway.

Surprisingly, a daily activation of GPR119 with OEA or other synthetic agonists, increases β-cell responsiveness in islets transplanted into STZ-induced diabetic mice (328).

The importance of GPR119 in the fat-induced incretin secretion is demonstrated by the impaired incretin signaling displayed by transgenic animals deficient for this protein only in PG expressing intestinal cells. Male and female mice, completely loose the GLP-1 response to an oral gavage of olive and corn oil (329).

More recently, it was reported that whole-body GPR119^−^knockout mice are protected from high-fat induced glucose intolerance and insulin insensitivity. Interestingly, the specific ablation of GPR119 only in β-cells does not affect glucose tolerance nor insulin secretion. In fact AR231453, a selective GPR119 agonist, improves glucose tolerance and insulin sensitivity in both WT and Gpr119^β*cell*−/−^, suggesting how insulin release is independent from pancreatic GPR119 but depends on gut incretin release (330).

Curiously, GPR119 activity appears to be directly dependent on the PYY receptor NPY1 (331). This phenomenon is independent of DPP-IV, the GLP-1 receptor, or the PYY related peptide NPY.

Furthermore, GPR40 also shows synergism with GPR119, mediating a more than additive GLP-1 response to triglycerides in the large intestine (263).

Agonism of GPR119 in both healthy or diabetic and obese mice, is known to improve glucose tolerance (90), or even prevent atherosclerosis in mice (332), while at the same time inducing the secretion of glucagon under low glucose levels avoiding hypoglycaemia (91); therefore since 2008, multiple agonists have been synthesized (239, 254, 333), as well as unimolecular dual DPP-4 inhibitors and GPR119 agonists (334). Despite the good results seen in rodents, species-specific pharmacology might be to blame (335).

Up to now all the prospective GPR119 agonists were plagued by low bioavailability, lack of efficacy and more importantly, cardiotoxicity which has stopped all human studies before any large scale Phase III clinical trials (239).

Despite the multiple failures, the compound DS-8500a is showing promising glucose lowering properties in Phase II clinical trials without any apparent toxicological issues in clinical trials (92).

## TGR5

Bile acids (BAs) are cholesterol-derived molecules produced in the liver and temporarily stored in the gallbladder. When food is ingested, BAs are released into duodenum to solubilize dietary lipids under the form of micelles, a necessary step for the maximization of the surface-to-volume ratio of fat droplets, aiding interface-acting lipases.

Indeed the release of lipids from micelles has directly proven to release GLP-1 and GIP via the FFAR1 in the duodenum (264).

This release of bile acids, mainly cholic (CA) and chenodeoxycholic (CDCA) acid derivatives, happens through the relaxation of the smooth muscle sphincter upon CCK signaling (336) or indirectly through a similar VIP action on the sphincter of Oddi (337).

Historically described as mere fat-solubilizing agents, these amphipathic compounds were recently recognized as key signaling molecules capable to modulate the host metabolism directly acting as ligands of intestinal GPCRs (101, 338, 339), or after being metabolized by the colonic microbiota into secondary bile acids, mostly deoxycholic and lithocholic acid(340).

The chemosensor believed to be the main receptor of bile acids is TGR5, also known as GPR131 or GPBAR1 among other names. This receptor has been reported to be expressed by colonic GLP-1-secreting enteroendocrine cells and pancreatic α-and β-cells (100, 101), with some controversy regarding the presence in murine islets (339).

TGR5 activity appears to not have been lost in type 2 diabetic humans whereby the infusion of CCK, or rectal taurocholate, causes GLP-1 and insulin release via the TGR5 axis in colonic L-cells and pancreatic β-cells respectively (341, 342).

This notion is in stark contrast to the well-known anti-diabetic properties of BAs sequestrants, (343) and some, have proven to elicit GLP-1 secretion via TGR5 mediated PC1/3 upregulation (344). A likely explanation is that the BAs bound to a sequestrant into the intestinal lumen can't be absorbed and hence travel more distally in the GI tract where the complexes are still capable to activate the TGR5 expressing colonic L-cells. Furthermore, the lower systemic levels of bile salts prompt the liver to produce more bile, which in turn feeds more TGR5 agonism into the colon (343).

This chemosensor is expressed by the pancreatic α-cells where its signaling activates Gs proteins and induces the secretion of GLP-1 directly through Epac proteins and indirectly via CREB mediated expression of Psck1, while in β-cells mediates insulin release [(100); Figure 3].

TGR5 is the target of different BAs, but the most potent endogenous agonist has shown to be lithocholic acid (LCA) and its taurine conjugates with activity at nanomolar concentrations (273, 339). Secondary bile salts, metabolized by the microbiota, exhibit less potency toward this receptor.

Despite this promising anti-diabetic activity of TGR5 mediate by GLP-1 (345), its pharmacological activation in diabetic patients has shown side effects at the level of gallbladder and heart, hampering its clinical use (346).

Another bile salts chemosensor is the nuclear farnesoid X receptor (FXR) (347) which activation, contrarily to TGR5, blocks the release of GLP-1 in the colonic L-cells (348), while in the liver induces glycogenesis helping to improve glucose homeostasis. This counterintuitive pharmacology has been confirmed *in vivo* whereby the administration of the FXR agonist GW4064 by mouth drives hyperglycaemia and obesity (349) while intraperitoneal injection exerts protection from it (350). Consistently, an indirect inhibition of intestinal FXR through microbiota modulation, or genetic deletion of intestinal FXR, corroborate this phenome displaying protection from high-fat diets induced obesity and fatty liver disease (351).

This could explain why bile acid sequestrants support a positive glucometabolic homeostasis. Indeed, the insoluble complexes of bile salts can activate lumen-facing TGR5 receptors, while they cannot cross plasma membranes to activate intra-cellular GLP-1-suppressant FXR receptors.

FXR is a very important receptor, part of a negative feedback in the liver, whereby the binding of bile salts, especially chenodeoxycholic acid, represses the *de-novo* synthesis of bile salts (352, 353). Indeed, there are multiple primary or secondary bile acid chemosensors in the liver (348, 354) or scattered along the gastrointestinal tract (355), where they ensure a direct negative feedback aiding detoxification (356) and protecting from hepatotoxicity and carcinogenicity displayed by some secondary bile salt such as lithocholic acid.

Accumulated evidence, indicate how bile acids are important modulators of the whole body metabolism, bridging the microbiome to the brain, likely being key signaling molecules in the pathogenesis of obesity and type 2 diabetes. Indeed remittance from diabetes experienced by RYGB or SG patients, has been attributed to the elevation of circulating bile acids (37, 38, 357), warranting further investigation, especially the development of gut-restricted TGR5 agonists (358).

## TRPV1 and the TRP channel family

The transient receptor potential vanilloid 1 (TRPV1) is a tetrameric non-specific cationic channel found in most of mammalian sensory neurons (359). Each of its constituting monomers crosses the plasma-membrane six times and both the N and C-term face the cytoplasmic side, where they make up 70% of the receptors' entire volume (360). This chemosensor, together with other 27 non-selective cationic channels, is part of a larger family named transient receptor potential (TRP) channel superfamily and is known to play an important role in the metabolic syndrome (361, 362).

TRPV1 is primarily activated by vanilloids and capsaicinoids including Capsaicin (360), eliciting the sensation of spiciness; multiple stress-related stimuli cause its activation and opening with subsequent membrane depolarization. For example cigarette smoke, excess of protons (pH < 5.9) (363), temperatures above 43° (360), certain animal toxins (364, 365), ATP (366) or even cannabinoids such as Anandamide (367) and cannabidiol (359, 368), are all stimuli known to activate this sensor. Indirect stimulation has also been demonstrated by bradykinin (366), NGF (366), PGE2 (369), PGI2 (369) and agonists of Protease-activated Receptors (PARs) (370).

TRPV1 has been shown to be expressed in the brain, β-cells (371), nociceptor C fibers, dorsal root ganglia, hepatocytes, spermatozoa (372), airway neurons (373), bladder and urothelium (374), blood vessels, and the whole gastrointestinal myenteric plexus (375), especially in colonic and rectal neurons (376). Consistently, TRPV1 is also found to be expressed by the murine enteroendocrine cell line model STC-1 and its agonism induces the release of GLP-1 *in vivo* (377).

This receptor has recently seen an increasing interest since its activation has been found to have pleiotropic beneficial metabolic effects (378).

Indeed, it has been known for more than a decade that capsaicin is capable to elicit a glucose-stimulated insulin release *in vivo* (379). A crossover study operated on 30 human healthy subjects (380), showed a slight increase in plasmatic GLP-1 and a slight decrease in ghrelin levels 30 min after a Capsaicin enriched meal (containing 1,030 mg of 80,000 Scoville heat units red pepper); Peptide YY changes were not statistically significant. Despite these promising results, TRPV1 knockout mice display contrasting phenotypes with the report of opposite phenotypes. One author describes an obese insulin and leptin resistant mouse (381), while another group report animal protected from diet-induced obesity (382).

Considering all the recent findings, drugs targeting TRPV1 would be beneficial for the management of obesity (383) metabolic syndrome (384) and type 2 diabetes (385). Nonetheless, considering the EECs receptome responsible for gut-peptide modulation, TRPV1 has received much less attention, with a yet largely unexplored physiology.

## The microbiota

Animals' GI tract is known to host a population of hundreds of different species of bacteria (386), viruses and fungi, estimated to equal in number the cells that constitute the human body (387). These microorganisms thrive in the colon's lumen, where they secrete small molecules ultimately affecting the host immunity (240) and metabolism (388).

The relative abundance of different microbial species is known to depend on the presence of specific nutrients (389); hence, considering that an imbalance in the microbiota correlates with chronic inflammation pathologies of the bowel, or even Type 2 diabetes, it is likely that dietary components indirectly influence the occurrence of these pathologies via the microbiota (390, 391).

The human colonic microflora is known to produce high concentrations of Short-Chain-Fatty acids (SCFAs), among other metabolites, from the anaerobic fermentation of dietary indigestible carbohydrates, or even derivatives of bile salts (389). In fact, the SCFAs Acetate, Propionate and Butyrate are the principal luminal anions in humans and other mammalian's colon (309, 392), with some inter-species variability. Rats show higher levels of fecal Acetate, 75 mM vs. human's 50 mM, Propionate, 27 vs. 11 mM and Butyrate, 16 vs. 5 mM respectively. On the other hand, surprisingly similarly to humans' colonic and fecal values, rumens of herbivores, such as sheep or cows, also contain high levels of acetate, propionate and butyrate, with reported concentrations of 65, 21, and 18 mM, respectively (308). These levels appear to be independent of dietary proteins or fibers; conversely, it is the caloric intake that affects the relative composition and concentrations of SCFAs (308). These metabolites have been found to target specific receptors among the repertoire expressed by the EECs, triggering a hormonal response. It is estimated that in humans almost all fermented SCFA are absorbed by the colonocytes and only 5% are excreted with stool, equivalent to 5–30 millimoles per day. Indeed, it is not practically feasible to measure intraluminal production fluxes of various metabolites *in vivo* in humans; therefore, most studies focus on the easiest but less informative quantification of fecal SCFA content (393).

Despite the most recent studies of transgenic and germ-free animals, it is still largely unknown by what degree hormones such as GLP-1, and all its related peptides, depend on the microflora, especially in pathologies such as type 2 diabetes.

Recent high-throughput pharmacogenomic studies have deepened our understanding of the molecular players in this human-microbiota relationship. Recently it was shown that a new class of N-acyl amides is produced by the microbiota, and target GPCRs expressed by the enteroendocrine cells, modulating GLP-1 expression and overall glucose metabolism. In particular, N-oleoyl serinol (N-OS) is described as a potent GPR119 agonist, acting in the lower micromolar range with twice the efficacy of the endogenous ligand OEA (394).

From the evolutionary perspective, dietary components, together with the microbiota-fermented products, have activated the enteroendocrine system for billions of years, since the dawn of metazoan. Considering the vast and continuous pool of metabolites produced and modulated by the microbiota, the distinction between orthosteric and allosteric ligand becomes blurred; different molecules are likely working in synergy to elicit a specific hormonal response.

Modulation of the microbiome has shown promising results in the treatment of type 2 diabetes. For example, recently it was reported that a rhubarb extract, Rhein, increasing the intestinal population of Bacteroidetes, mediates an increase in ileal GLP-1 producing cells, peripheral GLP-1(7-36)_NH2_ levels and improved glucose tolerance in diabetic db/db mice (395). Consistently, STZ-treated rats, are protected from oxidative and inflammatory stress when treated with Liraglutide, and *Bacteroides*, as well as *Lactobacilli* strain populations appear to be restored (396).

In the last decade, the scientific community has just started to unveil the molecular pathways produced by this long-lasting symbiosis. It appears that SCFAs not only induce the release of GLP-1, they also represent a mitogenic signal. Rats fed oligofructose, a substrate for the colonic microbiota which leads to higher SCFAs levels, possess an increased number of colonic L-cells (397). This has been confirmed *ex-vivo* in human and mouse small intestinal crypts organoids (398).

Other compounds such as bile salts and xenobiotics (399), are known to be metabolized and excreted by the microbiota, affecting the host physiology. Indeed, the pharmacokinetic and pharmacodynamics of any drug taken by mouth should be appraised considering the role of the microbiota, as the varied efficacy of some chemotherapeutics such as 5-FU has been proven to directly depend on this host-microbiota metabolism (400). Even though the anatomical intestinal rearrangement of RYGB and SG patients is known to affect the microbiota, this doesn't appear to result in a different bile acid metabolism in a rat model (401).

We are at the beginning of a new branch of medical practice, tailored not only to the single person genome, but also to the microbiome.

Future human studies will help us to better understand the big picture of this relationship, to hopefully provide mechanistic knowledge upon which new treatments could be created, such as microbiome-directed gene-therapies for the management of metabolic diseases.

## Conclusion and perspective

GLP-1R-independent signaling of GLP-1, its intra-islet axis, and its once-thought inactive metabolites, all represent new important additions to our understanding of this peptide in health and disease.

Omnivores' gastrointestinal tract has co-evolved in strict relationship with a dynamic microbiota and a complex seasonal and regional diet, resulting into a robust and flexible system tightly interconnected via multiple neuroendocrine axes with different organs.

In nature, dietary fats are scarce energy-dense nutrients primarily found in fish and meat. This evolutionary pressure over millions of years has shaped a system for the attentive sensation, assimilation and storage of precious bioactive molecules in all superior animals.

Sensation happens at multiple levels with a plethora of somewhat redundant intestinal receptors (402), specifically in the enteroendocrine cell system. This redundancy can be seen in transgenic animals, whereby the genetic absence of a single chemosensor doesn't always result in a phenotype, probably due to metabolic compensation from similar and overlapping pathways.

Virtually all macronutrients are absorbed in the small intestine, where maximal activity of the EECs is ensured, while the colonic and rectal GLP-1 secretion is enforced in response to secondary metabolites even hours after the meal ingestion. This pattern is disrupted in bariatric patients undergoing RYGB surgery, where a remodeled GI tract delivers more nutrients to the large intestine, and changes gut-secretome, including its microflora.

Attempts to mimic this altered meal processing, such as proximal blockage of nutrient absorption resulting in increased delivery of nutrients in the distal intestine, have shown some promising results in healthy and diabetic humans (403). Although this is more challenging with fats because dietary lipids require partial digestion by lipases to become efficient secretagogues (404, 405). However, distant delivery of free fatty acids, or even Oleoyl-Glycerol and sodium taurocholate have shown negligible effects on peripheral levels of GLP-1 or PYY, satiety and glucose tolerance (311, 406, 407). Similarly, distal delivery of the best known aminoacidic GLP-1-secretagogue, glutamine, has proven ineffective at ameliorating glucose tolerance in both healthy and diabetic subjects (407–409).

Furthermore, a recent report (410) examined the effect of RYGB on lean pigs, and indicates how it is the post-operative GLP-1 (9-36)_NH2_ levels that raise, while surprisingly the “active” (7-36)_NH2_ peripheral levels were reduced.

Indeed, most authors focus only on the peripheral levels of only one of these two peptide species, vastly excluding GLP-1(28-36) _NH2_ and (32-36)_NH2_ activity, rendering the overall understanding of each individual GLP-1 species, in both health and disease, difficult to discern.

Technical advances ELISA, capable to specifically dissect these peptide species locally and peripherally, will help us to shed new light into this complex physiology (411).

Conclusively, bearing in mind that insulinotropic or incretinotropic effects are not secondary to any single receptor modulation, whereby pools of different luminal stimuli act synergistically on tens of different chemosensors during their intestinal transit and absorption, while interacting with the microflora metabolism, rendering the restoration of a healthy physiology in diabetic patients with the pharmacological correction of a single axis, highly improbable.

The final dissection of the molecular axis causative of either metabolic syndrome will need more evidence regarding the localized and inter-neuronal physiology of GLP-1 in physiological and pathological statuses. To ultimately tease apart any possible cause from secondary events, species-specific biology will also need to be carefully dissected and interpreted.

## Author contributions

SP researched and interpreted all the data from available scientific literature on the PUBMED database, organized, wrote and revised the whole manuscript. SP also conceptualized and drew all the figures assembling the final formatted review. MF conceived, organized, wrote and revised the whole manuscript.

### Conflict of interest statement

The authors declare that the research was conducted in the absence of any commercial or financial relationships that could be construed as a potential conflict of interest.

## Footnotes

^1^http://press.novonordisk-us.com/2017-12-5-Novo-Nordisk-Receives-FDA-Approval-of-OZEMPIC-R-semaglutide-Injection-For-the-Treatment-of-Adults-with-Type-2-Diabetes
